# How Does African Swine Fever Virus Evade the cGAS-STING Pathway?

**DOI:** 10.3390/pathogens13110957

**Published:** 2024-11-02

**Authors:** Can Lin, Chenyang Zhang, Nanhua Chen, François Meurens, Jianzhong Zhu, Wanglong Zheng

**Affiliations:** 1 College of Veterinary Medicine, Yangzhou University, Yangzhou 225009, China; lc2068118712@outlook.com (C.L.); chenyangzhang43@outlook.com (C.Z.); hnchen@yzu.edu.cn (N.C.); jzzhu@yzu.edu.cn (J.Z.); 2 Comparative Medicine Research Institute, Yangzhou University, Yangzhou 225009, China; 3 Joint International Research Laboratory of Agriculture and Agri-Product Safety, Yangzhou 225009, China; 4 Jiangsu Co-Innovation Center for Prevention and Control of Important Animal Infectious Diseases and Zoonoses, Yangzhou 225009, China; 5 Swine and Poultry Infectious Diseases Research Center, Faculty of Veterinary Medicine, University of Montreal, Saint-Hyacinthe, QC J2S 2M2, Canada; francois.meurens@umontreal.ca; 6 Department of Veterinary Microbiology and Immunology, Western College of Veterinary Medicine, University of Saskatchewan, Saskatoon, SK S7N 5E2, Canada

**Keywords:** African swine fever virus, innate immunity, DNA sensing, cGAS, STING

## Abstract

African swine fever (ASF), a highly infectious and devastating disease affecting both domestic pigs and wild boars, is caused by the African swine fever virus (ASFV). ASF has resulted in rapid global spread of the disease, leading to significant economic losses within the swine industry. A significant obstacle to the creation of safe and effective ASF vaccines is the existing knowledge gap regarding the pathogenesis of ASFV and its mechanisms of immune evasion. The cyclic GMP–AMP synthase (cGAS)–stimulator of interferon genes (STING) pathway is a major pathway mediating type I interferon (IFN) antiviral immune response against infections by diverse classes of pathogens that contain DNA or generate DNA in their life cycles. To evade the host’s innate immune response, ASFV encodes many proteins that inhibit the production of type I IFN by antagonizing the cGAS-STING signaling pathway. Multiple proteins of ASFV are involved in promoting viral replication by protein–protein interaction during ASFV infection. The protein QP383R could impair the function of cGAS. The proteins EP364R, C129R and B175L could disturb the function of cyclic guanosine monophosphate-adenosine monophosphate (cGAMP). The proteins E248R, L83L, MGF505-11L, MGF505-7R, H240R, CD2v, E184L, B175L and p17 could interfere with the function of STING. The proteins MGF360-11L, MGF505-7R, I215L, DP96R, A151R and S273R could affect the function of TANK Binding Kinase 1 (TBK1) and IκB kinase ε (IKKε). The proteins MGF360-14L, M1249L, E120R, S273R, D129L, E301R, DP96R, MGF505-7R and I226R could inhibit the function of Interferon Regulatory Factor 3 (IRF3). The proteins MGF360-12L, MGF505-7R/A528R, UBCv1 and A238L could inhibit the function of nuclear factor kappa B (NF-Κb).

## 1. Introduction

African swine fever (ASF) is an acute hemorrhagic viral disease caused by the African swine fever virus (ASFV), affecting domestic pigs and wild boar [1]. ASF is known for its exceptionally high fatality rate; domestic pigs that contract highly virulent ASFV strains experience mortality rates reaching 100% [2]. ASF has rapidly spread worldwide and inflicted significant economic losses on the swine industry [3,4]. ASFV is the sole member of the Asfarviridae family, characterized by its enveloped icosahedral deoxyribovirus structure and shared structural, genomic, and replicative features with other nucleocytoplasmic large DNA viruses [5]. ASFV is a large, enveloped, double-stranded DNA (dsDNA) virus harboring a linear DNA genome ranging from 170 to 190 kb in size and encompassing over 150 open reading frames (ORFs) [6]. Numerous research findings have indicated a strong correlation between the virulence and pathogenicity of ASFV isolates and the immunomodulatory functions of ASFV genes [7]. ASFV encodes many proteins dedicated not only to virus assembly but also to restraining the innate immune response [8].

The innate immune system serves as the initial barrier of defense for the host against invading pathogens [9]. The presence of aberrant nucleic acids in the cytoplasm will be sensed by the innate immune system, which triggers a response from type I interferon (IFN-I) [10]. Cyclic guanosine monophosphate-adenosine monophosphate synthase (cGAS) detects cytoplasmic microbial DNA and endogenous DNA resulting from genomic instability, thereby initiating innate immunity and playing pivotal roles in antiviral defense as well as the pathogenesis of various diseases [11]. Upon dsDNA binding, cGAS produces 2′3′-cyclic-GMP-AMP (2′3′-cGAMP), a second messenger that binds to the ER resident stimulator of interferon genes (STING) to induce innate immune responses [12]. Upon binding 2′3′-cGAMP, STING undergoes translocation from the ER to the endoplasmic reticulum–Golgi intermediate compartment (ERGIC) and subsequently to the Golgi apparatus, which is reliant on the COP-II complex and ARF GTPases [13]. The trans-Golgi network (TGN) serves as a site where STING recruits and activates TANK-binding kinase 1 (TBK1), subsequently leading to the activation and nuclear translocation of interferon regulatory factor 3 (IRF3) and nuclear factor kappa B (NF-κB) [14]. The cGAS-STING DNA sensing pathway has been found to have a crucial function in stimulating IFN-I and inhibiting the replication of viruses, specifically for DNA viruses [15]. After internalization, the nucleic acid of ASFV is primarily detected by the cytoplasmic DNA sensor cGAS [16]. To evade the host’s innate immune response, ASFV produces numerous proteins that hinder the generation of IFN-I by counteracting the cGAS-STING signaling pathway through protein–protein interactions [17]. The past decade has witnessed numerous studies demonstrating the involvement of multiple proteins from ASFV in facilitating viral replication through protein–protein interactions with host factors during ASFV infection [16,18]. The objective of this article is to provide a comprehensive overview of the strategies employed by ASFV to impede the cGAS-STING pathway.

## 2. The Mechanisms of ASFV Suppress the cGAS-STING Pathway

### 2.1. ASFV Inhibits the cGAS-STING Pathway Through Impairing the Activity of cGAS

cGAS is located on chromosome 6q13 and encodes a protein consisting of 495 amino acids in swine. The structure of porcine cGAS consists of three distinct domains: a regulatory domain at the (N)-terminal region spanning amino acid residues 1 to 134, an NTase domain covering amino acid residues 135 to 305, and a Mab21 domain encompassing amino acid residues 238 to 495 [19]. The interaction between cGAS and DNA is sequence-independent. The cGAS protein efficiently penetrates the minor groove of DNA and interacts with the sugar–phosphate backbone through its positively charged surface and the zinc-ribbon domain of Mab21 [20]. In order to maintain consistent catalytic activity, cGAS must form a dimeric structure. The catalytic domain of cGAS contains two DNA-binding sites that encompass approximately 16–18 base pairs of DNA, effectively sandwiching the DNA strand between the dimer. By utilizing ATP and GTP as substrates, cGAS is able to synthesize the second messenger molecule, known as 2′3′-cGAMP, through its dsDNA-binding capability. Subsequently, this synthesized molecule binds and activates STING, which is located within the ER [21].

**Figure 1:**
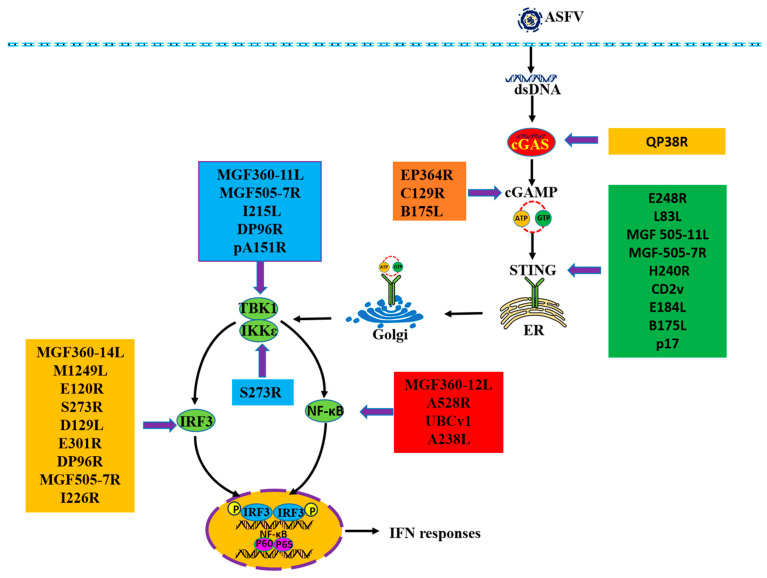
ASFV inhibits the cGAS-STING pathway by utilizing different viral proteins to target various signaling proteins. The viral proteins targeting cGAS-STING signaling proteins are shaded with different colors.

As an essential component in the cytosol, cGAS serves as a unique detector of DNA and plays a crucial role in combating DNA viruses. Therefore, various counteractive approaches against cGAS have been found in ASFV. The ASFV protein QP383R is an uncharacterized polypeptide consisting of 383 amino acids. Overexpression of QP383R could suppress the activation of IFN-I stimulated by dsDNA [19]. It has been indicated that the protein QP383R in ASFV could directly interact with cGAS and promote the palmitoylation of cGAS [19]. The cGAS protein possesses numerous sites for modification, allowing for regulation through a multitude of diverse post-translational modifications, encompassing phosphorylation, sumoylation, acetylation, ubiquitination and palmitoylation [22]. It has been suggested that the palmitoylation of cGAS could reduce the interaction between cGAS and double-stranded DNA, which could further inhibit the dimerization of cGAS [23]. Thus, ASFV QP383R could dampen IFN-I production by promoting cGAS palmitoylation (Figure 1).

### 2.2. ASFV Dampens the cGAS-STING Pathway Through Disturbing the Activity of 2′3′-cGAMP

Cyclic dinucleotides play pivotal roles as essential second messengers in both prokaryotes and eukaryotes, participating in a wide range of biological processes [24]. In mammalian cells, the primary form of CDN is 2′3′-cGAMP, which possesses a distinct 2′-5′ phosphodiester linkage produced by cGAS. 2′-3′-cGAMP (hereinafter referred to as cGAMP) is produced by the cytosolic DNA sensor cGAS in reaction to the existence of abnormal dsDNA in the cytoplasm, which is associated with microbial invasion or cellular damage [24]. The secondary messenger cGAMP plays a crucial role in the activation of STING, thereby initiating the production of IFN-I and pro-inflammatory cytokines that are indispensable for mounting effective immune responses against microbial invasion [25]. The asymmetric nature of cGAMP enables it to elegantly bind to two symmetric STING dimers with opposing orientations. The STING dimer contains a ligand-binding pocket where the cGAMP molecule attaches. This pocket has an uncharged base but is surrounded by positively and negatively charged residues on its walls, as well as interdomain interactions that involve multiple pairs of polar contacts [26]. The LBDs of dimeric STING adopt a butterfly-like structure in the cytosol, and binding of cGAMP induces a conformational change in the STING dimer. The alteration entails the closure of the ligand-binding pocket within the cytosol, followed by a subsequent 180° rotation of the LBD in relation to the transmembrane region [27]. The conformation likely facilitates the recruitment of coat protein complex II, thereby enabling the “exit” for STING to depart from the ER and traverse through COP-II vesicles towards the ERGIC and Golgi apparatus [26]. At the Golgi/ERGIC, STING recruits TBK1, thereby inducing activation of IRF3 and NF-κB [28].

Several studies have identified that the proteins EP364R, C129R and B175L of ASFV could act as specific negative regulators of cGAS-STING signaling through interacting with cGAMP [29,30] (Figure 1) (Table 1). It has been shown that ASFV EP364R and C129R could inhibit the activity of cGAS-mediated IFN-β promoter and reduce the cellular level of cGAMP [29]. Analyzing the sequence of EP364R showed that EP364R contains a cGAMP binding motif that allows it to compete with STING for binding cGAMP, and amino acids Y76 and N78 of EP364R are required for interaction with cGAMP [29]. Furthermore, study has also shown that the protein B175L could significantly inhibit DNA virus-induced IFN-β production and IFN-mediated signaling responses [30]. The protein B175L has demonstrated an ability to interact with cGAMP, thereby inhibiting the interaction between cGAMP and STING. The inhibition is attributed to the competitive interaction between the conserved zf-FCS motif of B175L and both cGAMP and the cyclic dinucleotide binding domain of STING [30].

### 2.3. ASFV Negatively Regulates the cGAS-STING Pathway Through Interfering with the Activity of STING

STING is a highly conserved cytoplasmic receptor in mammals that plays an indispensable role in the detection of cyclic dinucleotides synthesized by cGAS upon recognition of intracellular DNA [56]. STING plays a vital function in IFN production when it detects intracellular DNA or DNA pathogens, such as bacteria and DNA viruses [57]. It is indicated that the porcine STING is encoded by the TMEM173 gene and composed of 378 amino acids [58]. The STING protein is a dimeric transmembrane protein that localizes to the ER or Golgi apparatus. This unique location renders STING well poised to respond to intracellular organelle stress [59]. The STING protein is composed of four transmembrane helices in the N terminus, which are succeeded by the exquisite cytosolic CDN-binding domain (CBD) [60]. The presence of a flexible C-terminal tail in STING plays a crucial role in the recruitment and activation of TBK1 and IRF3 [61]. In the presence of cytosolic DNA, which binds to cGAS and triggers the production of cGAMP, STING dimerizes and subsequently translocates from ER to ERGIC or Golgi apparatus, and ultimately relocates to lysosomes for degradation [62]. After STING is transferred from ERGIC, it activates TBK1 to stimulate the production of IFNs and various cytokines.

Emerging evidence indicates that various proteins from ASFV have the potential to modulate STING, thereby inhibiting the cGAS-STING pathway and suppressing IFN-I production [18,33] (Figure 1) (Table 1). Firstly, it has been shown that proteins L83L, MGF505-11L and MGF505-7R of ASFV could inhibit the expression of STING or promote the degradation of STING [18,31,32]. The ASFV-encoded protein L83L, consisting of approximately 81 to 83 amino acids, is non-essential for viral activity but exerts a significant influence on the initial stages of viral replication. It has been suggested the L83L protein exerts a suppressive effect on the transcription of IFN-β and ISGs, while also inhibiting the phosphorylation of TBK1 and IRF3 [18]. L83L could promote the degradation of STING, and this degradation could be significantly reversed by the autophagy inhibitor 3-MA or proteasome inhibitor MG132, whereas the caspase inhibitor ZVAD did not demonstrate similar restorative effects [18]. ASFV MGF505-11L could inhibit the phosphorylation of TBK1 and IRF3 stimulated by the cGAS-STING pathway [31]. It has been indicated that MGF505-11L could interact with STING and cause the degradation of STING through the lysosomal, ubiquitin-proteasome and autophagy pathways [31]. MGF505-7R has been demonstrated to be the key virulence factor for ASFV [63]. It has suggested that MGF505-7R could inhibit the activation of IFN-β promoters mediated by the cGAS-STING pathway, and mediate the degradation of STING [32]. The degradation could be completely inhibited by the autophagosome inhibitor 3-MA but not by the lysosomal inhibitor NH4Cl or proteasome inhibitor MG132, which suggested that the STING was degraded by MGF505-7R through the autophagy pathway [32].

Secondly, it has been shown that the proteins H240R and CD2v of ASFV could inhibit the transportation of STING from ER to Golgi apparatus [33,34]. The H240R protein, a capsid protein of ASFV, was found to inhibit the production of IFN-I. It was shown that the protein H240R could interact with the N-terminal transmembrane domain of STING, which could affect the translocation of STING from ER to Golgi apparatus [33]. The protein CD2v of ASFV, encoded by the EP402R gene, is located around viral factories during ASFV infection, and it is required for the hemadsorption of red blood cells around ASFV-infected cells [34]. It has been indicated that the CD2v protein has the potential to interact with STING’s transmembrane domain, thereby impeding STING’s trafficking to the Golgi apparatus and subsequently hindering the functionality of the cGAS-STING signaling pathway [34].

Thirdly, it is also suggested that the proteins E184L and B175L could impair the dimerization and oligomerization of STING [30,35]. A study has shown that E184L could disrupt the formation of the STING-TBK1-IRF3 complex, resulting in a hindrance to STING phosphorylation and IRF3 dimerization as well as nuclear translocation [35]. E184L could interact with STING, leading to the inhibition of STING dimerization and oligomerization while preserving its puncta formation at the perinuclear region [35]. It was indicated that the 1-20 amino acid region of E184L is essential for E184L-STING interaction and blocking IL-1β and IFN-I production [35]. In addition, study has shown that the protein B175L could significantly inhibit the DNA virus-induced IFN-β production and IFN-mediated signaling responses [30]. It has been suggested that the protein B175L contains an MYM-type zinc finger domain with an FCS sequence motif. The conserved MYM-type Zinc finger with its FCS sequence motif enables B175L to function as a specialized inhibitor of the IFN-I response through direct interaction with STING [30]. The interaction between B175L and STING could affect the polymerization of STING [30].

Lastly, our previous study demonstrated that the protein p17 of ASFV is capable of inhibiting the cGAS-STING pathway by interacting with STING and disrupting the recruitment of TBK1 and IKKε. The p17 protein, which plays a vital role in cellular function, is present in both the protective capsid and the inner lipid envelope. It plays a vital role in the assembly and maturation of the icosahedral capsid, as well as ensuring overall virus viability due to its high abundance and essential nature [64]. The p17 protein of ASFV has the ability to suppress the cGAS-STING signaling pathway, thereby inhibiting the anti-HSV1 and anti-VSV responses. Furthermore, it has been proposed that the interaction between p17 and STING, as well as the subsequent inhibition of the cGAS-STING pathway, may rely on the crucial involvement of its transmembrane domain (amino acids 39–59) [36].

### 2.4. ASFV Suppresses the cGAS-STING Pathway Through Affecting the Activity of TBK1 and IKKε

The IKK family members are categorized into the canonical family members, namely IKKα and IKKβ, and the noncanonical family members, specifically TBK1 and IKK [65]. TBK1 and IKKε play critical roles in innate immunity by inducing IFN-I response. It is widely acknowledged that TBK1 plays a predominant role in the activation of IRF3 and NF-κB, as well as downstream cytokine production, induced by the cGAS-STING pathway [66]. However, multiple lines of evidence have demonstrated that STING can induce IFN production through the participation of both TBK1 and IKKε [66]. The domain composition of TBK1 and IKKε is similar, and they both share the same consensus phosphorylation modification for substrates. Both TBK1 and IKKε are capable of phosphorylating the C-terminal Ser/Thr rich regions of IRF3 and IRF7 [67]. Subsequently, the nuclear translocation of IRF3 and IRF7 leads to the induction of IFN-I. Previous studies have demonstrated that IKKε redundantly participates with TBK1 in both STING-induced IFN response and NF-κB response [68]. Our recent research revealed that porcine IKKε plays a crucial role in the induction of IFN-I response and antiviral activity within the porcine cGAS–STING pathway [66]. It appears that IKKε and TBK1 exhibit cooperative behavior in STING-induced IFN-I expression, while both demonstrate redundancy in the STING-mediated NF-κB response [66].

TBK1 and IKKε are vital for the induction of antiviral innate immune responses. Studies have indicated that several proteins encoded by ASFV could disturb the function of TBK1 and IKKε [37,38,39] (Figure 1) (Table 1). Firstly, it has been shown that the proteins MGF360-11L and MGF505-7R of ASFV promote the degradation of TBK1 [37,63]. The protein MGF360-11L effectively suppresses the activation of IFN-β and ISRE promoters activated by the cGAS-STING pathway, leading to a reduction in mRNA expression levels in IFN-β, ISG15, and ISG56 [37]. Studies have shown that the protein MGF360-11L could interact with TBK1 and promote the degradation of TBK1 through the cysteine, ubiquitin-proteasome and autophagy pathways [37]. Furthermore, study has also shown that the protein MGF505-7R could suppress the activity of IFN-β and ISRE promoters and the expression of IFN-I and ISGs activated by the cGAS-STING pathway [38]. MGF505-7R could interact with TBK1 and result in degrading TBK1 through the proteasome pathway [38].

Secondly, it has been shown that the proteins I215L, DP96R, A151R and S273R of ASFV could affect the post-translational modification of TBK1 or IKKε [39,40,41,46]. The tight regulation of TBK1 and IKKε involves various post-translational modifications, such as phosphorylation, sumoylation, ubiquitination, and acetylation [69]. The protein I215L, an exquisite viral E2 ubiquitin-conjugating enzyme, has been recognized as one of the most formidable suppressive agents in hindering the generation of IFN-I [39]. The protein I215L could enhance the interaction between RNF138 and RNF128 and promote RNF138 to degrade RNF128, which results in a decrease in K63-linked polyubiquitination of TBK1 [39]. Research has indicated that DP96R serves as a potential protein for immune evasion and plays a crucial role in the pathogenesis of domestic pigs [70]. DP96R has the potential to suppress the antiviral response induced by TBK1 and the phosphorylation of TBK1 triggered by cGAS-STING activation [40]. DP96R effectively suppressed the phosphorylation of TBK1 and subsequent IRF3 phosphorylation during cGAS-STING co-transfection [40]. A151R, a non-structural protein, has been identified as a thioredoxin and is indispensable for the replication and morphogenesis of the virus [71]. According to research, it has been found that A151R significantly suppressed the production of IFN-β triggered by the cGAS-STING signaling pathway [41]. A151R inhibits TBK1 K63-linked polyubiquitination and phosphorylation by promoting the degradation of E3 ligase TRAF6 [41].

Additionally, our previous study indicated that the enzymatic activity of S273R functions as a prominent suppressor of the cGAS-STING pathway by specifically targeting IKKε [42]. The cGAS-STING signaling pathway was significantly attenuated by S273R, which specifically targeted IKKε but not TBK1. Moreover, it was observed that S273R disrupted the interaction between IKKε and STING through its direct binding to IKKε [42]. Further, mutational analyses revealed that the enzymatic activity of S273R exerts an antagonistic effect on the cGAS-STING pathway, potentially influencing the sumoylation status of IKKε, which is necessary for its interaction with STING [42].

### 2.5. ASFV Suppresses the cGAS-STING Pathway Through Inhibiting the Activity of IRF3

The primary recognition of the IRF family of transcription factors lies in their pivotal role in regulating gene expression that governs IFN responses [72]. There exist a total of nine IRF proteins in mammals, which encompass IRF1, IRF2, IRF3, IRF4/PIP/LSIRF/ICSAT, IRF5, IRF6, IRF7, IRF8/ICSBP, and finally the one known as IRF9 or ISGF3γ [73]. The transcription factor IRF3 is constitutively expressed in various cell types, where it predominantly localizes to the cytoplasm in an inactive state [74]. The induction of IFN-I via the transcription factor IRF3 is considered a pivotal consequence of STING activation, driving immune response against DNA viruses. STING functions as a pivotal scaffold protein, facilitating and augmenting the phosphorylation of IRF3 by TBK1 [75]. The phosphorylation of IRF3 by TBK1 facilitates the dimerization and nuclear translocation of IRF3, thereby inducing gene expressions of IFN-I and ISGs, as well as various inflammatory mediators, chemokines, and pro-apoptotic genes [76].

It has been shown that various proteins of ASFV can modulate the activity of IRF3 through promoting the degradation of IRF3, impairing the interaction between IRF3 and TBK1, and interfering with the interaction between IRF3 and p300, blocking the transportation of IRF3 from cytoplasm to cell nucleus, and impairing the activation of IRF3 [43,44,45,46,47] (Figure 1) (Table 1). Firstly, studies have shown that the proteins of MGF360-14L and M1249L could promote the degradation of IRF3 [43,44]. MGF360-14L, a non-structural protein of ASFV, has the ability to hinder the activation of IFN-β promoter induced by cGAS-STING signaling. MGF360-14L was also observed to decrease the protein level of IRF3 and facilitate its degradation through ubiquitin-mediated proteolysis [43]. The interaction between MGF360-14L and IRF3 was demonstrated to result in the destabilization of IRF3 through the facilitation of E3 ligase TRIM21-mediated K63-linked ubiquitination of IRF3 [43]. The protein M1249L is an exquisite late-phase expression protein composed of a remarkable 1249 amino acids [77]. Acting as the fundamental component of capsid framework assembly, the M1249L capsid protein establishes extensive intermolecular interactions with other capsid proteins to facilitate the formation of the capsid framework and dictate its size [77]. The ectopic expression of M1249L protein exerts a significant inhibitory effect on the IFN-β promoter activity induced by the cGAS-STING pathway [44]. The study demonstrated that the interaction between M1249L and IRF3 resulted in the initiation of IRF3 degradation through the lysosomal pathway [44].

Secondly, it has been shown that the proteins E120R and S273R of ASFV could disturb the interaction between IRF3 and TBK1, and the protein D129L could interfere with the IRF3-p300 interaction [45,46,47]. E120R, a structural protein of ASFV, exerts a crucial role in inhibiting the phosphorylation of IRF3 and suppressing the production of IFN-I by impeding the recruitment of IRF3 to TBK1 [45]. The interaction between IRF3 and TBK1 may be impaired by E120R, with amino acids 72 and 73 of E120R playing a crucial role in suppressing this interaction [45]. S273R could inhibit the production of IFN-I by interacting with IRF3, which could disrupt the association between TBK1 and IRF3 [46]. Furthermore, D129L could suppress the induction of IFN-β through interfering with the interaction of IRF3-p300 [47]. The histone acetyltransferase p300, acting as a transcriptional co-activator, is commonly recruited to transcriptional enhancers where it exerts regulatory control over gene expression via chromatin acetylation. IRF3 dimers could bind to p300, stimulating chromatin acetylation and gene expression of the antiviral IFN-I [78]. D129L could interact with p300, exerting an inhibitory effect on IFN-β induction, and the interaction between pD129L and p300 relies on the indispensable presence of the HrcA domain [47].

Thirdly, it has been shown that the proteins E301R, DP96R and MGF505-7R of ASFV could block the nuclear translocation of IRF3 [38,48,49]. The E301R protein serves as a potent negative regulator, effectively suppressing the production of IFN-I and undermining the innate antiviral immune response during ASFV infection [48]. E301R interacts with amino acid region 1–200 of IRF3, leading to the inhibition of nuclear translocation of IRF3 induced by the cGAS-STING pathway [48]. DP96R disrupts the interaction between IRF3 and a critical karyopherin (KPNA) binding site, thereby impeding the nuclear translocation of IRF3 through interference with KPNA-IRF3 binding [49]. MGF505-7R, a member of the multigene family 505, exerted potent inhibitory effects on the production of IL-1β and IFN-β [63]. It was shown that the interaction between MGF505-7R and IRF3 impedes the intracellular trafficking of IRF3 from cytoplasm to cell nucleus [38].

Lastly, study has shown that the I226R protein possesses the ability to impede the activation of IRF3, thereby effectively suppressing the expression of IFN [51]. The I226R protein, encoded by the viral I226R gene, has a molecular weight of approximately 27 kD. Its expression can be observed during both early and late phases of viral infection, potentially influenced by factors such as host characteristics, virus strain, and challenge dose [79]. The activation of the IFN-β promoter and the ISRE reporter induced by cGAS-STING expression was hindered by the presence of the I226R protein [51]. The I226R protein effectively attenuated the host’s innate immunity, likely by exerting a negative regulatory influence on IFN expression and subsequently reducing the levels of crucial ISGs [51]. It has been reported that the I226R gene of ASFV plays a crucial role in determining the virulence level, and removing this specific gene significantly reduces the pathogenicity of SY18 in pigs [79].

### 2.6. ASFV Suppresses the cGAS-STING Pathway Through Inhibiting the Activity of NF-κB

The NF-κB transcription factor plays a crucial role in coordinating inflammatory responses induced by cGAS-STING during viral infections. The NF-κB pathway is widely recognized as the primary regulator of inflammation, while the cGAS-STING pathway has been shown to effectively induce NF-κB activity in response to viral infections [80]. The STING-NF-κB response is effectively mediated through a redundant mechanism involving TBK1 and IKKε. The TBK1 and IKKε complexes coordinate canonical NF-κB responses and facilitate the production of pro-inflammatory cytokines. The presence of TBK1 is unnecessary for the activation of NF-κB responses induced by STING in both human and mouse immune cells, as well as in vivo [81]. Recently studies have also revealed that activating of NF-κB could in turn enhance the STING responses through altering microtubule trafficking [82]. The activation of NF-κB pathway could induce the depolymerization of microtubules, which could inhibit STING trafficking to lysosomes for degrading [82]. This leads to increased levels of activated STING that persists for a longer period of time, thereby prolonging and increasing STING signaling [82].

It has been shown that MGF360-12L, MGF505-7R/A528R and UBCv1 could affect the function of NF-κB through blocking the nuclear translocation of NF-κB [52,53,54] (Figure 1) (Table 1). The competitive inhibition of the interaction between NF-κB and nuclear transport proteins by MGF360-12L may potentially impede the nuclear translocation of NF-κB [52]. Importin α, also known as karyopherin α (KPNA), functions as the nuclear import receptor that specifically recognizes both classical monopartite and bipartite nuclear localization signals (NLSs) [83]. The nuclear import of NF-κB was impeded by MGF360-12L, effectively suppressing the interaction between p65 and KPNA2, KPNA3, and KPNA4. Consequently, the transcriptional activity of NF-κB was inhibited, thereby preventing the host’s antiviral response [52]. The MGF505-7R/A528R protein, a prominent member of the polygene family of ASFV, has been demonstrated to effectively suppress IFN-β production by specifically targeting the NF-κB signaling pathway [53]. MGF505-7R/A528R can suppress the NF-κB signaling pathway, including p65 phosphorylation and nuclear translocation [53]. It was shown that MGF505-7R/A528R could interact with p65 through the ANK repeat domains of A528R and RHD of p65 [53]. UBCv1 successfully inhibited the movement of p65 into the nucleus following cytokine stimulation, which is a critical step in NF-ĸB signaling [54]. ASFV ubiquitin-conjugating enzyme UBCv1 is able to mitigate inflammatory signaling by inhibiting NF-κB activation [54].

It has been shown that the A238L protein could affect the function of NF-κB through inhibiting the binding of NF-κB to DNA [55]. The protein A238L is present in both the nucleus and the cytoplasm in ASFV-infected cells [55]. The A238L protein possesses the ability to impede the activation of the NF-κB transcription factor and suppresses the phosphatase activity of calcineurin [55]. The presence of A238L did not result in an observed inhibition of nuclear translocation for NF-κB p50 and p65 subunits. The nuclear localization of A238L may exert a suppressive effect on the binding of NF-κB to DNA [55].

## 3. Conclusions and Future Perspective

**Figure 2:**
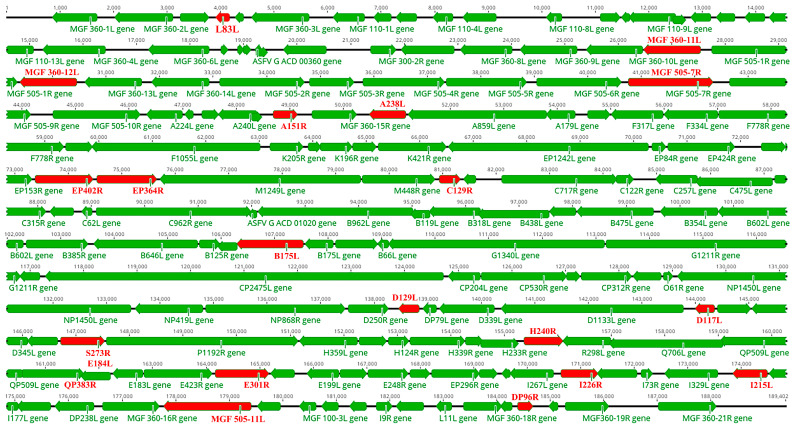
The genome organization of ASFV-SY18 is depicted, illustrating the arrangement of open reading frames (ORFs). ORFs that encode proteins inhibiting the cGAS-STING pathway have been highlighted in red.

ASFV encodes a large number of proteins that could impede the generation of the IFN-I response by blocking the cGAS-STING pathway in order to avoid the host’s innate immune response (Figure 2). The protein QP383R could impair the function of cGAS through promoting the palmitoylation of cGAS. The proteins EP364R, C129R and B175L could disturb the function of cGAMP through competing with STING to bind cGAMP, reducing the cellular level of cGAMP or disturbing the interaction between cGAMP and STING. The proteins E248R, L83L, MGF505-11L, MGF505-7R, H240R, CD2v, E184L, B175L and p17 could interfere with the function of STING through inhibiting the expression of STING, promoting the degradation of STING, inhibiting its transportation from ER to Golgi apparatus, impairing the dimerization and oligomerization of STING or interfering with STING’s ability to recruit TBK1 and IKKε. The proteins MGF360-11L, MGF505-7R, I215L, DP96R, A151R and S273R could affect the function of TBK1 and IKKε through promoting the degradation of TBK1, reducing the K63-linked polyubiquitination of TBK1, inhibiting the phosphorylation of TBK1, suppressing the polyubiquitination and phosphorylation of TBK1, or affecting the sumoylation of IKKε. The proteins MGF360-14L, M1249L, E120R, S273R, D129L, E301R, DP96R, MGF505-7R and I226R could inhibit the function of IRF3 through promoting the degradation of IRF3, impairing the interaction between IRF3 and TBK1, interfering with the interaction between the IRF3 and p300, blocking the nuclear translocation of IRF3, or impairing the activation of IRF3. The proteins MGF360-12L, MGF505-7R/A528R, UBCv1 and A238L could inhibit the function of NF-κB through blocking the nuclear translocation of NF-κB and disturbing NF-κB’s ability to bind DNA.

Since the introduction of the virus to Georgia in 2007, and subsequent spread across Europe and Asia, only genotype II ASFV has been detected [84,85]. Therefore, most studies have explored the mechanisms of ASFV evading the cGAS-STING pathway using genotype II ASFV as the research subject. However, since 2023, studies have indicated that several naturally occurring recombinants of genotype I and genotype II ASFV were identified in China, Vietnam and Russia [84,85,86,87]. The recombinant viruses identified exhibit high lethality and transmissibility in pigs, rendering the genotype II virus-based live attenuated vaccine ineffective against these variants. Facing the situation that ASFV is recombining, several crucial and meaningful questions naturally arise: are these proteins mentioned above evolutionarily conserved? Did all the proteins from different strains have the same function in immune evasion? Is it possible that the same protein in different strains has different functions in immune evasion? However, whether proteins from different strains have the same structure and function is still unclear. Thus, these recombinant strains should be taken as important research objects for investigating the mechanisms of ASFV evasion of immune systems in the future.

cGAS is shown to be a shuttle protein transported between the cytoplasm and nucleus. Within the cytoplasm, cGAS functions as a DNA sensor, eliciting IFN and inflammation responses. When transporting to the nucleus, the activity of cGAS could be inhibited through interacting with histones [88]. cGAS is exported into the cytoplasm by CRM1 in response to DNA stimulation [89]. The transportation of cGAS is dependent on the pathogenic infection. However, the effects of ASFV on the transporting and distributing of cGAS are not known. Thus, it is recommended that studies should be designed to explore the effects of ASFV on the transporting and distributing of cGAS. cGAS is regarded as one of the most critical cytosolic DNA sensors in vertebrates. Many studies have focused on the mechanisms by which ASFV evades the cGAS-STING pathway. Excepting cGAS, there are several other DNA sensors including AIM2, IFI16, DDX41 and ZBP1 that also exert significant roles in recognizing and preventing pathogenic microbes [90]. However, there are still huge gaps in the knowledge of ASFV interactions with other DNA sensing pathways. Thus, it is worth investigating the relationships between ASFV and other DNA sensing pathways. Additionally, it has been reported that infection with ASFV could actively lead to cell apoptosis and formation of apoptotic bodies (ApoBDs), which can be utilized by ASFV for infection and cell–cell transmission [85]. However, the exact regulatory mechanisms by which ASFV induces and hijacks ApoBDs are still unclear. It is predicted that in the future, related research about interactions between ASFV and ApoBDs will become a trend.

Since the ASF outbreak, scientists from many nations have been working on attenuated ASFV vaccines. The main challenge in developing an ASFV vaccine is the limited understanding of the molecular mechanisms of complex ASFV proteins and host antiviral responses. Investigating the functions of ASFV proteins in the immune system is of significance for developing attenuated vaccines in the future. Thus, more studies should be designed to explore the interaction between ASFV and the innate and adaptive immune systems.

## Figures and Tables

**Table 1:** Molecular mechanisms of ASFV regulating the cGAS-STING signaling pathway. Note: the red shade mainly targets cGAS; the orange shade mainly targets cGAMP; the yellow shade mainly targets STING; the green shade mainly targets TBK1/IKKε; the cyan shade mainly targets IRF3; and the blue shade mainly targets NF-κB.

No.	Gene/Protein	Target	Molecular Mechanism	Ref
1	QP383R	cGAS	Promoting the palmitoylation of cGAS	[19]
2	EP364R	cGAMP	Competing with STING to bind cGAMP	[29]
3	C129R	cGAMP	Reducing the cellular level of cGAMP	[29]
4	B175L	cGAMP	Disturbing the interaction between cGAMP and STING	[30]
5	L83L	STING	Promoting the degradation of STING	[18]
6	MGF 505-11L	STING	Promoting the degradation of STING	[31]
7	MGF-505-7R	STING	Promoting the degradation of STING	[32]
8	H240R	STING	Inhibiting the transportation of STING	[33]
9	CD2v	STING	Inhibiting the transportation of STING	[34]
10	E184L	STING	Impairing the dimerization and oligomerization of STING	[35]
11	B175L	STING	Impairing the dimerization and oligomerization of STING	[30]
12	p17(D117L)	STING	Interfering with STING’s ability to recruit TBK1 and IKKε	[36]
13	MGF360-11L	TBK1	Promoting the degradation of TBK1	[37]
14	MGF505-7R	TBK1	Promoting the degradation of TBK1	[38]
15	A238L(I215L)	TBK1	Reducing the K63-linked polyubiquitination of TBK1	[39]
16	DP96R	TBK1	Inhibiting the phosphorylation of TBK1	[40]
17	A151R	TBK1	Suppressing the polyubiquitination and phosphorylation of TBK1	[41]
18	S273R	IKKε	Affecting the sumoylation of IKKε	[42]
19	MGF360-14L	IRF3	Promoting the degradation of IRF3	[43]
20	M1249L	IRF3	Promoting the degradation of IRF3	[44]
21	E120R	IRF3	Impairing the interaction between IRF3 and TBK1	[45]
22	S273R	IRF3	Disrupting the association between TBK1 and IRF3	[46]
23	D129L	IRF3	Interfering with the interaction between the IRF3 and p300	[47]
24	E301R	IRF3	Blocking the nuclear translocation of IRF3	[48]
25	DP96R	IRF3	Blocking the nuclear translocation of IRF3	[49]
26	MGF505-7R	IRF3	Blocking the nuclear translocation of IRF3	[50]
27	I226R	IRF3	Impairing the activation of IRF3	[51]
28	MGF360-12L	NF-κB	Blocking the nuclear translocation of NF-κB	[52]
29	MGF505-7R	NF-κB	Blocking the phosphorylation and nuclear translocation of p65	[53]
30	UBCv1	NF-κB	Blocking the nuclear translocation of p65	[54]
31	A238L(I215L)	NF-κB	Inhibiting NF-κB’s ability to bind DNA	[55]
